# Analysis of the Influence of the Dynamic Characteristics of an Optical Bench on Optical Mechanical System Imaging Under Vibration Conditions

**DOI:** 10.3390/s25041268

**Published:** 2025-02-19

**Authors:** Yijian Wang, Ping Jia, Ping Wang, Zhongyu Liu, Yupeng Zhang, Lu Sun

**Affiliations:** 1 State Key Laboratory of Dynamic Optical Imaging and Measurement, Changchun Institute of Optics Fine Mechanics and Physics, Chinese Academy of Sciences, Changchun 130033, China; 2 University of Chinese Academy of Sciences, Beijing 100049, China

**Keywords:** optical bench, dynamic characteristics, vibration conditions

## Abstract

The imaging processes of optoelectronic devices are affected by vibration in the transportation platform, which can cause image shaking and blurring. Nowadays, devices often solve problems of image shaking and blurring using motion rotors. However, there is relatively little research on the influence of optical fixtures themselves under vibration conditions. This article analyzes the influence of sinusoidal vibrations on the MTF of an imaging process, pointing out the randomness of imaging effects under conditions of low-frequency vibration. To address the issue of low-frequency vibration effects, an analysis of the designs, and experimental verification, of a specific optical system mount were conducted to verify the influence of the mount’s own properties on imaging under random vibration conditions, providing a basis for the design of future optical mechanical systems.

## 1. Research Background and Significance

With the expansion of the application fields of modern optical systems, a large number of optical devices are being loaded on motion platforms such as cars, airplanes, and satellites. The mobile carrier optical system has high operability, flexible maneuverability, and strong concealability. It can adjust its position and attitude in real time according to an observed object and optical target, and can enter various complex, dangerous and hostile areas to work, greatly expanding potential ranges of information acquisition and transmission of optical systems. However, fixed optical systems have limited capabilities in this regard. Even so, sports platform environments are subject to a large amount of vibration, which can affect the operation of precision optical systems. For example, when the optical tracking equipment on a vehicle platform is subjected to vibration, the target surface image will experience severe shaking, and the tracking target may even be lost; When the optical detection equipment on an airborne platform is subjected to high-frequency vibration, it will produce image plane shaking and blurring. Therefore, studying the impact of vibration on imaging quality and quantitatively analyzing it is of great significance for the design of optomechanical structures and image processing [1].

As the basic form of vibration, sinusoidal vibration has been extensively studied by many scholars both domestically and internationally, but most studies have been limited to theoretical analysis and simulation. Hadar et al. proposed two methods for solving the dynamic transfer function of sinusoidal vibration: (1) based on frequency domain analysis, the MTF can be derived from modulation changes in the sinusoidal target after imaging through the system; (2) based on an analysis method in spatial domain, the line spread function is obtained by equating the point spread function (LSF) of the optical system with the probability density function (PDF) of the vibration over the exposure time [2,3,4,5].

At present, many articles on the stability of optical axes analyze the stability ability. On the one hand, by installing an optoelectronic platform on a two-dimensional or three-dimensional rotating frame and stabilizing the frame, the visual axis of the optical load can be stabilized. On the other hand, various isolation techniques are used to attenuate the vibration energy transmitted from the transporter to the optoelectronic platform. However, there is limited research on the influence of the stiffness characteristics of the structural system of optical devices after axis stabilization has occurred, with regard to the stability of the optical axis [6,7,8,9,10].

The optical bench is the installation benchmark of the optical system, and its own characteristics have a significant impact on the performance of the optical system. To reduce the performance of the optical socket, materials with lower linear expansion coefficients and higher stiffness values are usually used. Traditional optical socket materials include aluminum alloy, titanium alloy, and carbon steel. When considering special design requirements, such as the need for a highly lightweight system, magnesium alloy materials can be used. For systems that are highly sensitive to temperature, if the main mirror is made of SiC, the optical fixture holder can be made of Invar material that matches its linear expansion coefficient. Related scholars have applied aluminum-based silicon carbide (SiC/Al) materials to the designs of optical sockets based on their properties, and analyzed the first-order fundamental frequency, from 110 Hz to 240 Hz, through simulation. Another group of scholars designed a T-shaped turret light fixture base made of magnesium alloy material, and improved the structure to achieve a displacement of 4 μm under normal-gravity conditions. Table 1 is a preliminary analysis of commonly used optical socket materials and their advantages and disadvantages [11,12,13,14,15].

Previous studies only analyzed the performance of the optical pedestal itself, including deformation under gravity, stress distribution, and basic frequency simulation, and did not analyze its performance under vibration conditions, nor did it analyze the relationship between the dynamic performance under vibration conditions and the index of the optical imaging system. In this paper, the influence of vibration on the optical transfer function (MTF) is studied. Through the simulation analysis of the optical pedestal under static and dynamic conditions, and combined with experimental verification, the influence of the dynamic characteristics of an optical pedestal under vibration on the imaging performance of an optical system is studied.

## 2. The Influence of Vibration on MTF of Optoelectronic Devices

### 2.1. Impact Analysis of Vibration on Image Quality Degradation

In the imaging process, the relative motion between a sensor and an object, the optical phase difference, the image sensor, and other factors cause image distortion or deformation, referred to as image degradation. According to refs. [16,17,18], image degradation can be divided into motion blur degradation and noise degradation. The degradation model can be expressed as:

(1) g(x)=h(x)×f(x)+n(x)

where f(x) is the original image, h(x) is the point spread function, n(x) is the noise quotation mark, and g(x) is the degraded image.

During the exposure period of the camera, vibration of the optical system will cause received light energy to be dispersed, and the light energy distribution PSF (point spread function), which describes the light energy distribution in the process of image motion, will change accordingly.

The calculation formula for the PSF under sinusoidal vibration is:

(2) $$h(x)=\frac{1}{\pi }\frac{1}{\sqrt{D^{2}+x^{2}}}$$

The MTF that can evaluate the capabilities of the imaging system and the image quality can be obtained by Fourier transform and modulus of Formula (2). The calculation formula is:

(3) $$MTF(f)=\left|\frac{1}{t_{e}}\int_{t_{s}}^{t_{s}+t_{e}}\exp[-2j\pi fx(t)]dt\right|$$

where $t_{e}$ is the exposure time, $t_{s}$ is the initial exposure start time, and f is the spatial frequency.

In the imaging process of optoelectronic devices, the transfer function MTF is the most representative parameter indicator. Therefore, variations in MTF under vibration conditions can reflect the impact of vibration on the imaging of optoelectronic devices.

In the imaging process of optoelectronic devices, image motion can take different forms, including the forms of uniform linear motion, low-frequency vibration sinusoidal motion, and high-frequency vibration sinusoidal motion. MTF decreases with increasing spatial frequency, indicating a decrease in contrast at higher spatial frequencies. When the spatial frequency is high, the MTF of the system will drop below the output threshold function of the optical system, and the image of the system will become unobservable at that time. The maximum spatial frequency of the system MTF within the output threshold function range of the optical system is $f_{\max}$.

Observing the relative motion between an object and an optical system can cause image blurring, which can be caused by linear motion or reciprocating motion in the form of a sine function.

### 2.2. MTF of Linear Motion

The degradation of image quality caused by image plane motion can be analyzed through the following methods.

If the motion causes the object image to move linearly at a constant velocity v on the image focal plane, then for the exposure time $t_{e}$, the spatial size of the diameter D of the object image forming a fuzzy, irregular circle on the image focal plane is $vt_{e}$. To find the MTF of the image motion, we need to know the contrast between the intensity patterns of the image and the object. As a simple mathematical model, an image with a sinusoidal brightness pattern can be represented as follows:

(4) $$i(x)=B_{0}+B_{m}\cos2\pi fx(t)$$

Among them, f is spatial frequency, x(t) is the motion function of the *X*-axis in the spatial coordinate system, $B_{0}$ and $B_{m}$ are constants.

Therefore, the model for contrast (MC) without motion changes is as follows:

(5) $$MC_{0}=\frac{B_{m}}{B_{0}}$$

And if there is linear motion in the image:

(6) $$x(t)=x_{0}+vt$$

The formula for system brightness is as follows:

(7) $$i(x,t)=B_{0}+B_{m}\cos2\pi f(x_{0}+vt)$$

The exposure at any point is directly proportional to the average intensity within the exposure time $t_{e}$ interval:

(8) $$\bar{i(x,t)}=\frac{1}{t_{e}}\int_{0}^{t_{e}}\left[B_{0}+B_{m}\cos2\pi f(x_{0}+vt)\right]dt=B_{0}+B_{m}\frac{\sin(\pi fvt_{e})}{\pi fvt_{e}}\cdot \cos2\pi f(x_{0}+\frac{vt_{e}}{2})$$

Therefore, the new pattern has the same shape as the original pattern, but the image quality is determined by the speed.

According to the definition, the contrast of the (motion state) on the image plane is:

(9) $$MC_{i}=\frac{B_{m}\sin\pi fvt_{e}}{B_{0}\sin\pi fvt_{e}}=\frac{B_{m}}{B_{0}}\sin c(\pi fvt_{e})$$

And adjustment of the contrast function, MCF, to be equal to the sine wave response, MTF, is obtained through Equations (2) and (6):

(10) $$MTF=MCF=\frac{MC_{i}}{MC_{0}}=\left|\sin c(\pi fvt_{e})\right|$$

Among them, f is spatial frequency; when $fvt_{e}=1$, the equation is equal to 0.

At this point, the blur radius of the image $vt_{e}$ is equal to the reciprocal of the spatial frequency $f_{r\max}$.

When the spatial frequency is higher than ${\left(vt_{e}\right)}^{-1}$, the spatial range of the fuzzy radius is smaller than $vt_{e}$. Due to the fact that the fuzzy radius is smaller than the true minimum fuzzy radius at this time, such a situation, where the frequency is higher than the spatial frequency $f_{r\max}$, does not exist.

### 2.3. MTF of Sinusoidal Motion

Sinusoidal image motion needs to be given special consideration in optoelectronic device analysis due to its generation being caused by mechanical vibrations caused by the engines of airplanes and various transportation vehicles. In the design process of optoelectronic devices, sinusoidal vibration is the most important disturbance affecting image quality, and it is usually attenuated through appropriate design during the design process. The effect of sinusoidal motion on image quality change is determined by the ratio of exposure time $t_{e}$ to the period $T_{0}$ of the sinusoidal vibration motion. In the process of analyzing the impact of vibration, it is necessary to distinguish between the following two situations:

1. Low-frequency vibration: Exposure time is shorter than the period of vibration ($t_{e}<T_{0}$),

2. High-frequency vibration: Exposure time is longer than the period of vibration ($t_{e}>T_{0}$).

#### 2.3.1. High-Frequency Vibration

High-frequency vibration can generally be defined as one or more complete vibration periods falling within the exposure period. At this moment, the following is true: 

The analysis method is similar to that of uniform motion, and the motion function is:

(11) $$x(t)=D\sin(\frac{2\pi t}{T_{0}}+\varphi )dt\left(0\leq t\leq T_{0}\right)$$

The probability density function of its displacement is:

(12) $$f_{x}\left(x\right)=\frac{1}{n}\times \frac{n}{\pi \sqrt{D^{2}-x^{2}}}=\frac{1}{\pi \sqrt{D^{2}-x^{2}}}$$

Thus, we bring Formula (11) into Formula (3), and write the integral function in the Bessel series form, as:

(13) $$\begin{array}{ll} MTF(f,t_{s})= & \left|\frac{1}{t_{e}}\int_{t_{s}}^{t_{s}+t_{e}}\left[J_{0}(2\pi fD)+2\sum_{k=1}^{+\infty }J_{2k}(2\pi fD)\cos(\frac{4K\pi }{T_{0}}t)\right]\right.dt\\ & -\left.j\frac{1}{t_{e}}\int_{t_{s}}^{t_{s}+t_{e}}2\sum_{k=1}^{+\infty }J_{2k-1}(2\pi fD)sin(\frac{4K\pi }{T_{0}}t)dt\right| \end{array}$$

where $J_{n}$ is Bessel function of order *n*. In the formula, two infinite series converge in [0,+∞), and Formula (13) can be simplified as:

(14) $$\begin{array}{ll} MTF(f,t_{s})= & \left|J_{0}(2\pi fD)+2\sum_{k=1}^{+\infty }J_{2k}(2\pi fD)\frac{\sin\left(2k\pi \frac{t_{e}}{T_{0}}\right)}{2k\pi \frac{t_{e}}{T_{0}}}\cos\left(2k\pi \frac{2t_{s}+t_{e}}{T_{0}}\right)\right.\\ & -2j\left.\sum_{k=1}^{+\infty }J_{2k-1}(2\pi fD)\frac{\sin\left(\left(2k-1\right)\pi \frac{t_{e}}{T_{0}}\right)}{\left(2k-1\right)\pi \frac{t_{e}}{T_{0}}}\sin\left(\left(2k-1\right)\pi \frac{2t_{s}+t_{e}}{T_{0}}\right)\right| \end{array}$$

When the exposure time is an integral multiple of the angular vibration period, $t_{e}=nT_{0}$. At this time, according to Formula (13), the series term is 0, and as such,

(15) $$M_{s}(f)=\left|J_{0}(2\pi fD)\right|$$

Among them, D is the maximum amplitude.

**Figure 1:**
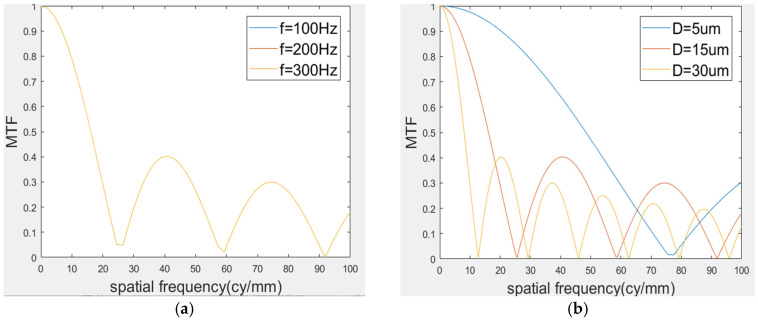
The MTF Curve: (**a**) same amplitude at different frequencies, (**b**) same frequency at different amplitudes.

It can be seen from Formula (15) that when the exposure time is an integral multiple of the vibration period, the MTF of the imaging system is independent of the vibration period and the initial exposure time, and only related to the vibration amplitude *D*. Taking the detector with an exposure time of 20 ms as an example, when the image plane amplitude is 15 μm and the initial exposure time is any time, the vibration frequencies are 50 Hz, 100 Hz, 150 Hz, and 200 Hz, respectively, as shown in Figure 1. The vibration frequency is 50 Hz, the initial exposure time is any time, the image vibration amplitude increases from 3 μm to 12 μm, and the MTF curve changes as shown in the figure. It can be seen that when the vibration frequency changes, the MTF curve basically does not change. When the vibration amplitude increases, the attenuation speed of the MTF curve gradually accelerates.

#### 2.3.2. Low-Frequency Vibration

**Figure 2:**
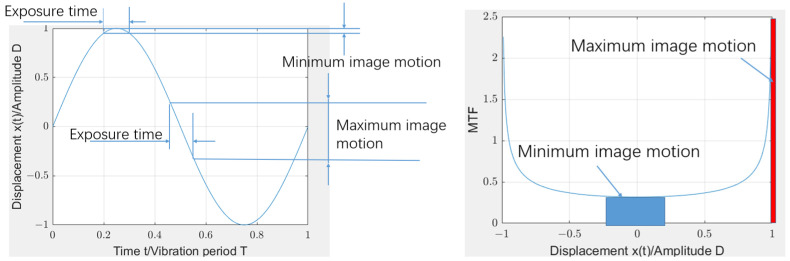
MTF calculation of low-frequency vibration using the density function of high-frequency vibration displacement.

The image motion caused by low-frequency vibration has a relatively long period $T_{0}$, which is longer than the exposure time. The image blur caused by low-frequency vibration only occurs during a part of the cycle, unlike imaging in high-frequency vibration, which occurs throughout the entire vibration cycle. The image blur caused by low-frequency vibration ($t_{e}<T_{0}$) is a random process. In this case, the imaging time for a given exposure time is random. As shown in Figure 2, there are two extreme cases of low-frequency vibration, where the minimum displacement occurs within the exposure time, centered on the mechanism point of the vibration period, and the MTF is approximately equal to 1. The maximum displacement occurs within the exposure time centered on the zero point, and its transfer function can be approximated by linear motion, i.e.,

(16) $$f_{MTF}=\left|\sin c(\pi fd_{\max})\right|$$

Due to the randomness of exposure time, there is no clear analytical expression for the MTF of low-frequency vibration.

Based on the low-frequency vibration conditions, the following conclusions can be drawn:

When imaging exposure occurs at the extreme value for vibration, blur is minimized, and when the exposure occurs at the center, image blur is maximized. In any case, the shorter the exposure time, the smaller the blur radius. The maximum and minimum fuzzy radii are as follows:

(17) $$d_{\min}=D\left[1-\cos\left(\frac{2\pi }{T_{0}}\right)\left(\frac{t_{e}}{2}\right)\right]$$

(18) $$d_{\max}=2D\sin\left[\left(\frac{2\pi }{T_{0}}\right)\left(\frac{t_{e}}{2}\right)\right]$$

Both low-frequency and high-frequency vibrations can affect the MTF of the system, and the specific impact is directly related to the exposure period and the position of the exposure occurring in the vibration period. The shorter the exposure time, the smaller the blur radius of the image.

**Figure 3:**
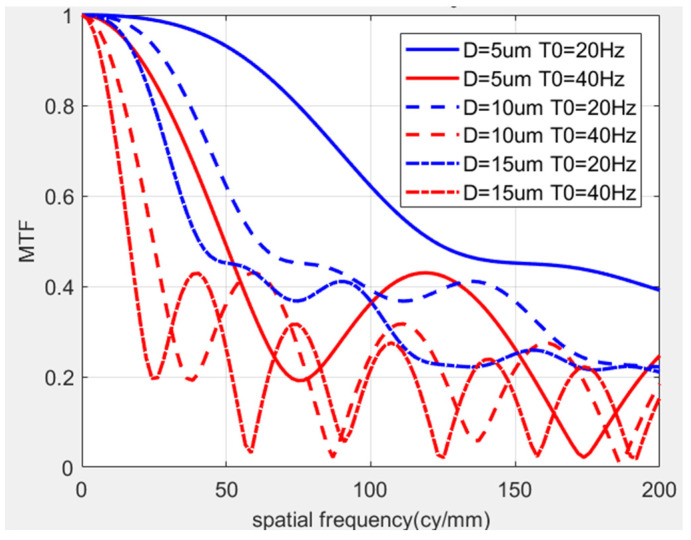
MTF curve at different frequencies and different amplitudes.

We set the initial exposure time to 0, the exposure time to 20 ms, the amplitudes to 5, 10, and 15 μm, and the vibration frequencies to 20 Hz and 40 Hz, respectively. The corresponding MTF curve is shown in Figure 3. When the vibration frequency is set thus, it can be seen that the amplitude and frequency of low-frequency vibration will affect the imaging quality. The MTF curve attenuates with increases in vibration amplitude, and the MTF decreases with increases in vibration frequency at the same amplitude.

## 3. Optical Bench Design

In common aperture optoelectronic devices, the optical tool-holder is the load-bearing component of the optical equipment, and all optical and imaging devices are installed on the optical tool-holder. Especially at present, many optoelectronic devices are adopting a common aperture design. By combining visible light and infrared light into one aperture, the device’s aperture and system focal length can be increased under the same envelope size, ultimately improving the device’s observation distance, resolution, and other indicators. Large aperture and long focal length optical systems have created increasing demand for optical equipment with stable frames, including the servo systems of such equipment and the stiffness of stable frames. As with the installation structure of the optical load, the design indicators of the optical tool-holder also need to be correspondingly improved.

### 3.1. Optical System Design

**Figure 4:**
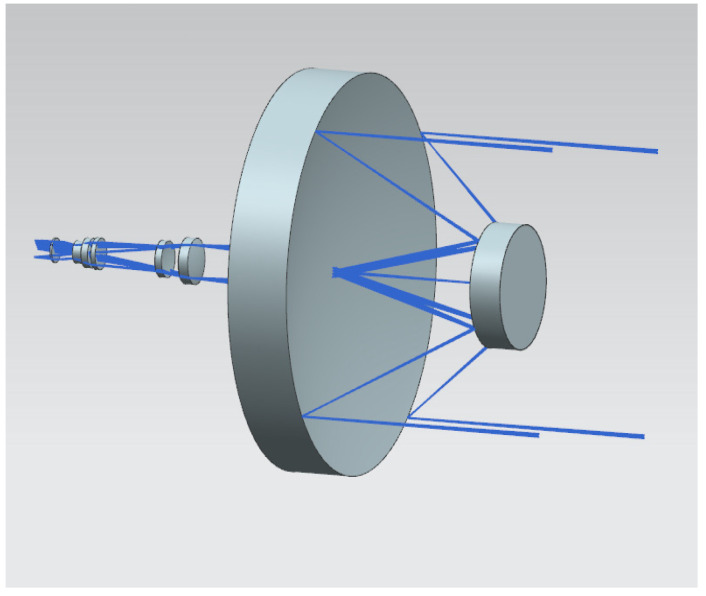
Light path diagram.

The primary and secondary mirror images of the optical equipment, as well as the visible light path, are shown in the following Figure 4, using a coaxial two-mirror card structure. The diameter of the main mirror is 175 mm, the focal length is 850 mm, and the field of view angle is 0.58° × 0.32°.

The pixel size of the visible light detector is 4.5 μm × 4.5 μm, with a pixel count of 4608 × 2176.

According to the calculation, the angular resolution corresponding to each pixel movement of the detector is 5.3 urad.

**Figure 5:**
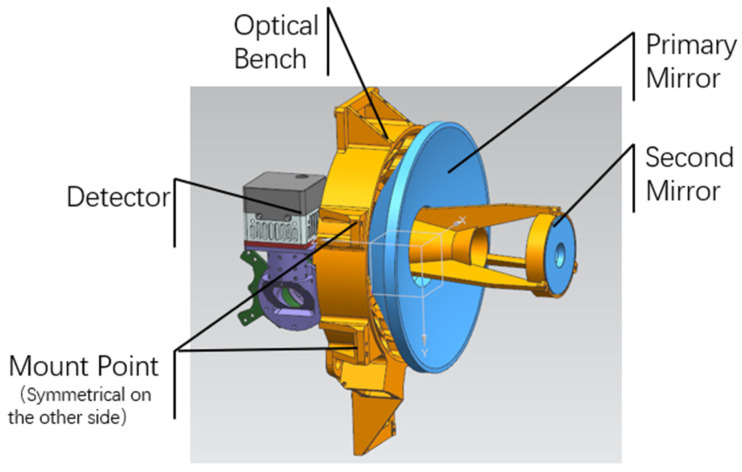
Structural model.

The models of the optical bench, primary and secondary mirrors, and visible light path are shown in Figure 5. In the design process of optical equipment, there are meant to be four installation points for the optical tool-holder and the external stable frame, as shown in Figure 5. The installation points for the optical bench are located on both sides of the main mirror to ensure the strength and rigidity of its installation position. The visible light path is located behind the main mirror, and can be adjusted to maximize the parallelism error of the optical axis.

### 3.2. Static Analysis of Optical Mechanical Structure

In order to quantify the structural deformation of the plate-shaped optical tool-holder under load-bearing conditions, it is necessary to conduct finite element static simulation analysis on the optical tool-holder. The optical tool-holder designed in this paper adopts titanium alloy, and the material properties are shown in Table 2.

In the pre-processing stage of the finite element analysis, the numerical model of the optical fixture base is simplified to obtain an idealized model. We unify the idealized model with the actual model, and add attributes such as mass and elastic modulus to the idealized model according to the actual model.

**Figure 6:**
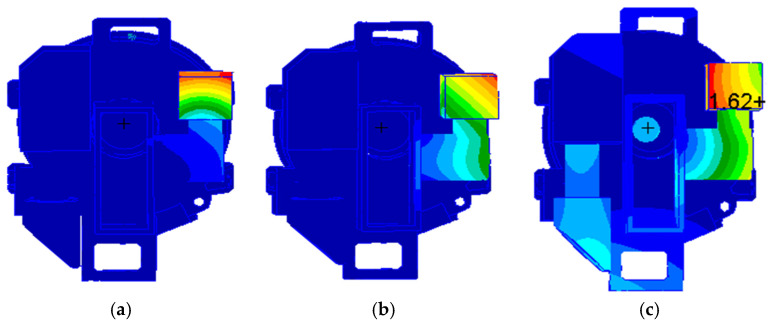
The vibration mode: (**a**) first order frequency; (**b**) second order frequency; and (**c**) third order frequency.

The simulation analysis results show that the first three fundamental frequencies of the visible light detector position are shown in Table 3. The vibration mode is shown in Figure 6.

**Figure 7:**
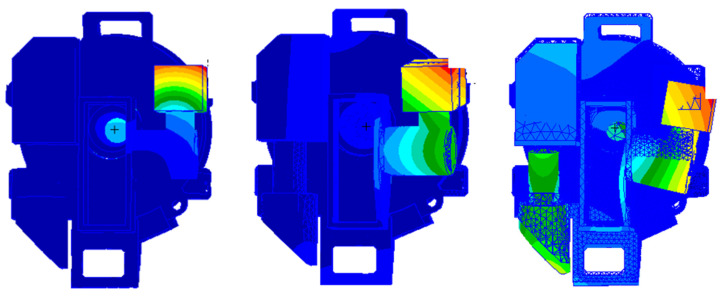
Displacement changes under the influence of 1 g gravity in three directions.

The static displacements in the *X*-axis, *Y*-axis, and *Z*-axis, under the action of gravity, are 5.61 μm, 5.22 μm, and 1.47 μm, respectively, as is shown in Figure 7. In the simulation analysis, it can be seen that the displacement of the optical bench under the action of gravity is about 1/10 that of the detector, so it can be considered as a rigid body in the analysis.

### 3.3. Random Vibration Analysis of Optical Mechanical Structure

**Figure 8:**
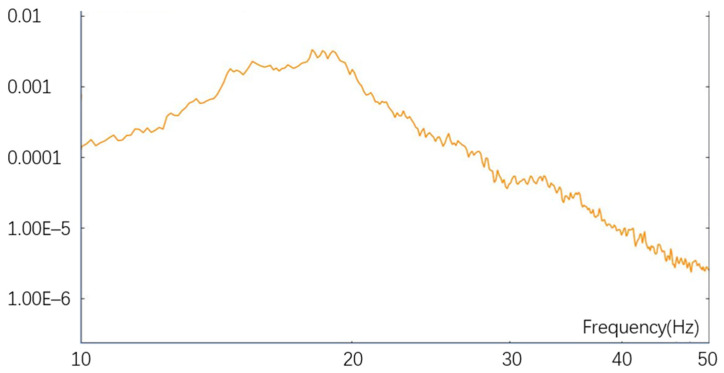
Measured curve after shock absorber.

**Figure 9:**
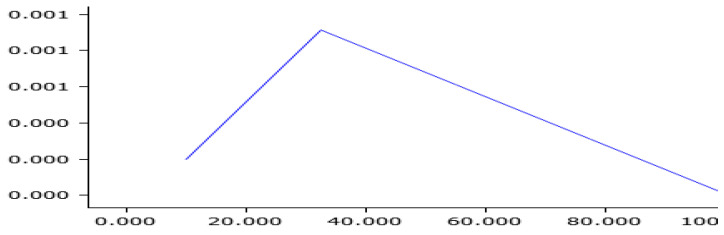
The simulation input curve.

**Figure 10:**
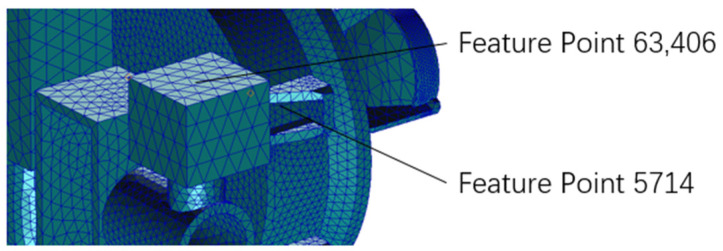
Location of feature points.

**Figure 11:**
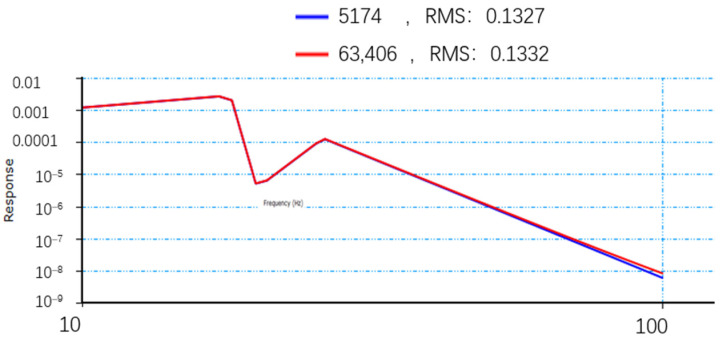

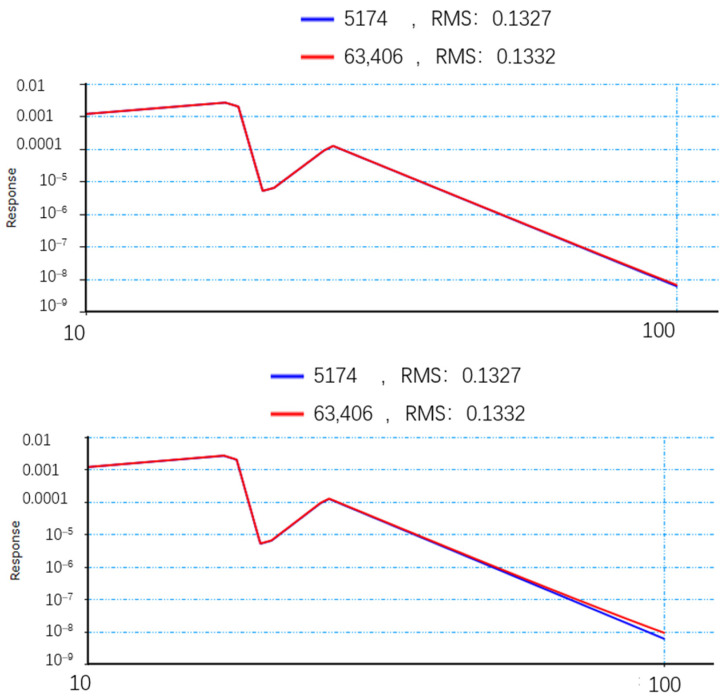
Triaxial vibration curve of feature points.

Due to the fact that optical loads are installed inside optoelectronic devices and undergo vibration transmission through a designed damping system, the random vibration analysis of the optical system must use vibration curves that have been tested in practice, as shown in Figure 8. The simulation input curve is shown in Figure 9. We select the feature points shown in Figure 10. The selection principle for feature point 1 is based on the maximum vibration position in the modal simulation analysis, while feature point 2 is selected as an approximately stationary point on the optical fixture seat. The vibration curve of feature points is shown in Figure 11.

### 3.4. Simulation Analysis Results

**Figure 12:**
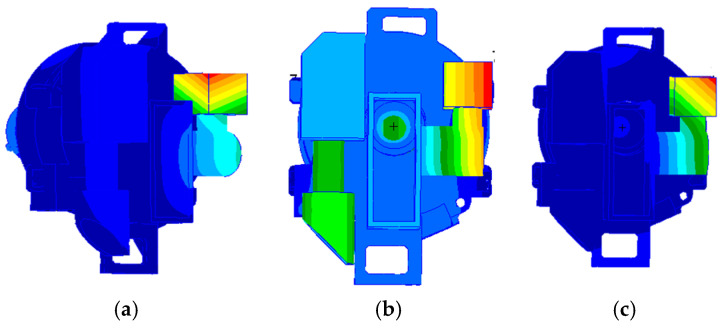
Maximum displacement point model of random vibration models: (**a**) X-direction vibration model; (**b**) Y-direction vibration model; (**c**) Z-direction vibration model.

According to the simulation analysis results for *X*-axis random vibration, by exporting the feature point data of each curve, the maximum difference between the two feature points can be found to occur at a frequency point of 20 Hz, as shown in Figure 12a. At this time, the static displacement difference between the detector and the optical tool-holder is about 13.2 μm, the pixel size of the detector is 4.5 μm, and the displacement between the detector and the optical tool-holder is 3 pixels.

According to the simulation analysis results for *Y*-axis random vibration, by exporting the feature point data of each curve, the maximum difference between the two feature points was found to occur at a frequency point of 25 Hz, as shown in Figure 12b. At this time, the static displacement difference between the detector and the optical tool-holder is about 8.2 μm, the pixel size of the detector is 4.5 μm, and the displacement between the detector and the optical tool-holder is 1.8 pixels.

According to the simulation analysis results for *Z*-axis random vibration, by exporting the feature point data of each curve, the maximum difference between the two feature points is found to occur at frequency point 25, as shown in Figure 12c. At this time, the static displacement difference between the detector and the optical fixture holder is about 42.6 μm, the pixel size of the detector is 4.5 μm, and the displacement between the detector and the optical fixture holder is 9.5 pixels. However, according to the figure, the *Z*-axis displacement occurs in the direction of the optical axis extension, and focusing adjustment occurs during the vibration process.

## 4. Experimental Result Verification

**Figure 13:**
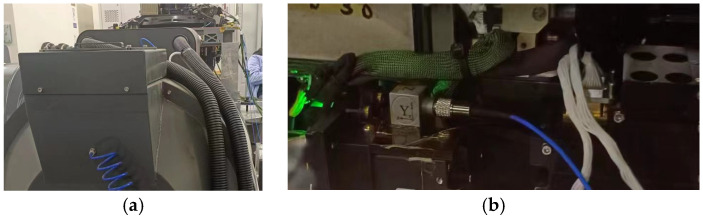
Picture of vibration test: (**a**) vibration table, (**b**) sensor adhesive point.

**Figure 14:**
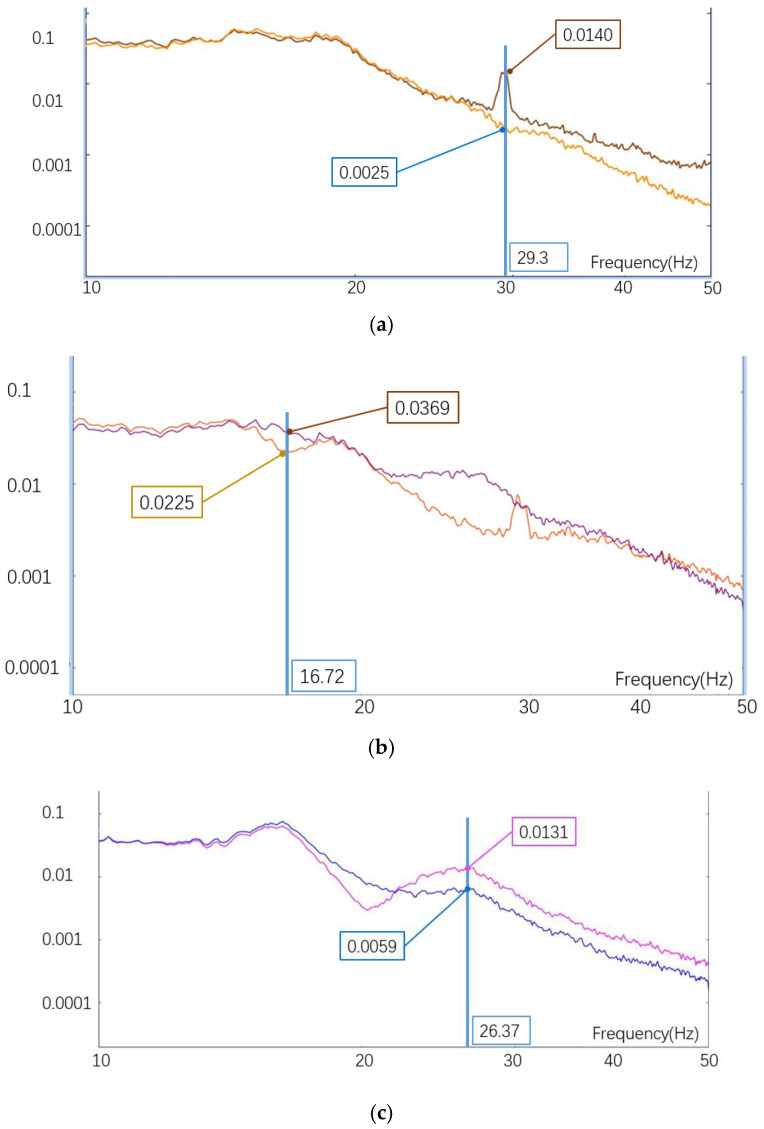
Random vibration test result curve: (**a**) *X*-axis displacement curve; (**b**) *Y*-axis displacement curve; (**c**) *Z*-axis displacement curve.

The simulation analysis results were verified through experiments, and the vibration test images are shown in the following figure, Figure 13a. The adhesive position of the sensor is shown in Figure 13b. The vibration input curve is shown in Figure 8, with the same input curve for all three directions. The vibration displacement curves in the three directions are shown in Figure 14. The following conclusions can be drawn from the images and corresponding data results: (1) The difference between the maximum displacement points of the X-direction vibration curve is 12.5 μm, with a frequency of 29 Hz. (2) The maximum displacement difference value for the Y-direction vibration curve collection is 7.2 μm, with a frequency of 26 Hz. (3) The maximum displacement difference value for the Z-direction vibration curve collection is 40.2 μm, with a frequency of 27 Hz. According to the output curve and analysis results, it can be seen that there is a certain error between the simulation analysis results and the actual measurement values. Reasons for analysis: ① Due to the use of quality substitution method in the simulation analysis process, not all components were modeled and simulated. ② In the actual process, screw connections are used between installed components, the damping of which cannot be simulated, resulting in certain errors in the results, but the overall trend is certain.

**Figure 15:**
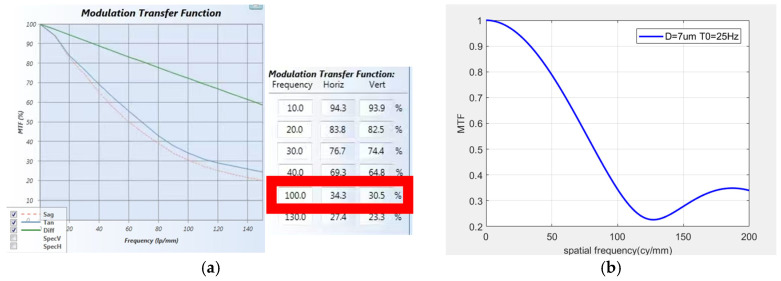
*X*-axis MTF curve: (**a**) screenshot of actual detection curve, (**b**) screenshot of simulation curve.

**Figure 16:**
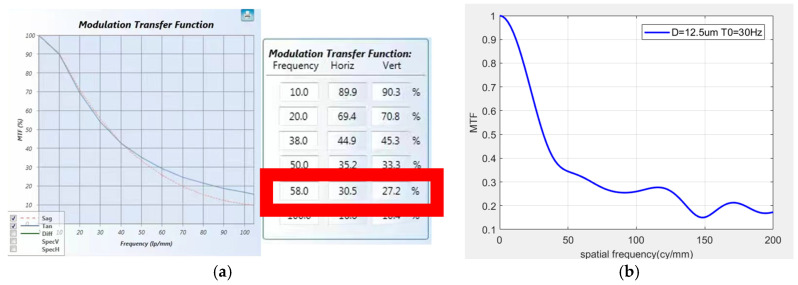
*Y*-axis MTF curve: (**a**) screenshot of actual detection curve, (**b**) screenshot of simulation curve.

**Figure 17:**
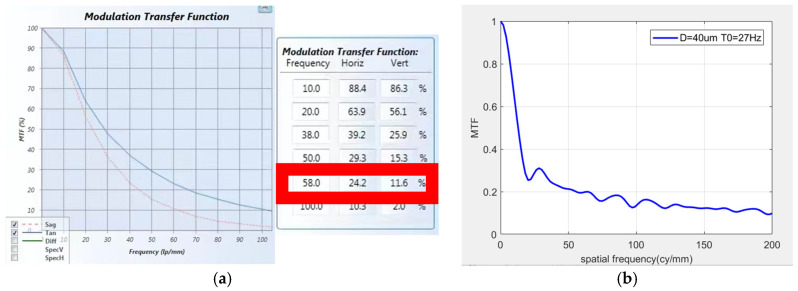
*Z*-axis MTF curve: (**a**) screenshot of actual detection curve, (**b**) screenshot of simulation curve.

The MTFs corresponding to the image at corresponding frequencies were detected, and MTF curves in three directions were captured. Screenshots of the transfer function were taken during the vibration process, and the results are shown in Figure 15, Figure 16 and Figure 17. Compare the theoretical transfer function calculated by the transfer function calculation formula with the actual one. According to the actual measurement values, the transfer function curves were calculated for amplitudes of 12.5 μm at 30 Hz, 7 μm at 25 Hz, and 40 μm at 27 Hz. The transfer functions at a spatial frequency of 100 cy/mm were calculated to be 25.93%, 34.34%, and 13.5%, respectively. The MTF values at a spatial frequency of 100 cy/mm were 22.7%, 30.5%, and 11.6%, respectively. The errors between the actual results and the theoretical calculation values are 12.4%, 11.2%, and 14.1%, respectively. This is because the transmission models of the light source and detector in the theoretical calculation process are both ideal models. However, in the actual results, due to differences in the performance of various optical components in the measurement optical system from the ideal model, there is a certain error between the actual results and the theoretical calculations. The error between the theoretical MTF model calculated in this paper and the actual measurement model is within an acceptable range.

## 5. Conclusions

The optical bench holder plays an important role in the design of common aperture optical systems, and its performance under its own dynamic conditions has an impact on the optical mechanical system, which cannot be isolated by servo stabilization. This article analyzes the influence of the performance of the optomechanical system on optical imaging under vibration conditions, and theoretically calculates the motion performance of the optomechanical system under high-frequency and low-frequency vibration conditions. By designing and analyzing the optical tool-holder in a visible light optical system with a main mirror diameter of 175 mm, a focal length of 850 mm, and a detector pixel of 4.5 μm, the simulation analysis results were found to be basically consistent with the experimental results. As such, the problem of image quality degradation caused by relative displacement between an optical tool-holder and a detector in the practical application of high-performance optical systems is solved, providing a basis for the design of future optical systems.

## Figures and Tables

**Table 1:** Analysis of Materials Used for Optical Bench.

Materials	Advantages	Disadvantages
2A12	low density low price easy processing	high coefficient of linear expansion low elastic modulus
TC4	high elastic modulus low coefficient of linear expansion	high density high price
4J32	coefficient of expansion matched with optical mirror materials	high density high price difficult processing
45	high elastic modulus low price easy processing	high density large coefficient of linear expansion
ZK61	low density	low elastic modulus high price
SiC/Al	low density high elastic modulus	difficult processing high price

**Table 2:** Analysis of material attributes in simulation.

Material	Elastic Modulus	Density	Poisson’s Ratio
TC4	101.5 GPa	4510 kg/m^3^	0.33
SiC	450 GPa	3200 kg/m^3^	0.31

**Table 3:** Simulation analysis of fundamental frequency results.

Vibration Mode	
First order frequency	245 Hz
Second order frequency	269 Hz
Third order frequency	553 Hz

## Data Availability

Due to the project being in the research phase, all raw data will be uploaded to the website after the project is completed and approved by the organization.
